# Periconceptional Folate Supplementation in Women after Bariatric Surgery—A Narrative Review

**DOI:** 10.3390/nu13051557

**Published:** 2021-05-05

**Authors:** An-Katrien Vynckier, Dries Ceulemans, Greet Vanheule, Paulien De Mulder, Mieke Van Den Driessche, Roland Devlieger

**Affiliations:** 1 Metagenics Europe, Edward Vlietinckstraat 20, 8400 Oostende, Belgium; an-katrien.vynckier@metagenics.eu (A.-K.V.); greet.vanheule@metagenics.eu (G.V.); mieke.vandendriessche@metagenics.eu (M.V.D.D.); 2 Department of Development and Regeneration, KU Leuven, Herestraat 49, 3000 Leuven, Belgium; dries.ceulemans@uzleuven.be; 3 Department of Obstetrics and Gynaecology, University Hospitals Leuven, Herestraat 49, 3000 Leuven, Belgium; 4 Department of Obstetrics and Gynaecology, Ghent University Hospital, Corneel Heymanslaan 10, 9000 Gent, Belgium; Paulien.DeMulder@ugent.be; 5 Department of Obstetrics, Gynaecology and Reproduction, St-Augustinus Hospital, Oosterveldlaan 24, 2610 Wilrijk, Belgium

**Keywords:** bariatric, pregnancy, folate, 5-MTHF, vitamin B9, periconception

## Abstract

The prevalence of obesity is increasing globally, and along with it, there is a growing number of patients opting to undergo bariatric surgery to treat this condition. Whilst it has many advantages, bariatric surgery is known to induce micronutrient deficiency, with possible deleterious effects on overall health. This topic becomes even more relevant during pregnancy, where deficiencies can also affect the developing fetus, possibly being the cause of an increase in congenital anomalies. Most notably amongst these micronutrients is folate, or vitamin B9, which plays an essential role in development, gene expression and genomic stability. As insufficient levels of folate are associated with neural tube defects in the fetus, preventing and treating folate deficiencies during pregnancies after bariatric surgery is a relevant issue. Unfortunately, folate supplementation recommendations for bariatric patients who wish to become pregnant are not clear. In this narrative review, we discuss whether the recommendations for the general population are still valid for bariatric patients. Furthermore, we discuss the role of folate in the human body, folate status in both non-bariatric and bariatric patients, the various types of folate that are available for substitution and the risk associated with over-supplementation.

## 1. Introduction

The prevalence of obesity is increasing globally, with reports indicating that the number of patients afflicted with this condition has nearly tripled since 1975 [1]. Obesity is associated with multiple comorbidities such as diabetes and cardiovascular disease, but also complications related to reproduction such as infertility, congenital malformations and various pregnancy complications [2,3]. Due to the impact of this condition on overall health and quality of life, the number of patients seeking treatment increases each year [4]. Patients with Grade III obesity (BMI > 40 kg/m^2^) or Grade II obesity (BMI > 35 kg/m^2^) with associated comorbidities are eligible for bariatric surgery. These types of surgery have been found to be the most effective and long-term solution, not only treating the excess weight, but also the related afflictions [5,6]. As more than 85% of patients undergoing these surgeries are women, many of whom are of reproductive age, and bariatric surgery improves fertility, pregnancies after bariatric surgery are becoming increasingly common [7].

Although bariatric surgery can reduce the risk for obesity-related pregnancy complications, there is also an increased risk for adverse outcomes due to the surgery itself [8,9]. Prominent amongst these is the possibility of micronutrient deficiencies [10]. One such micronutrient is folate, or vitamin B9, which is essential for DNA and RNA synthesis, cell multiplication and protein synthesis. As the demand for folate increases during pregnancy to sustain the growth and development of the fetus, deficiencies can occur more quickly and can have deleterious effects [11]. As such, supplementation of folate is of the utmost importance to prevent complications in the mother (anemia or neuropathy) and child (congenital abnormalities) [8,11]. In this narrative review, we discuss uptake and function of folate in the human body, the prevalence of deficiencies in surgical and non-surgical pregnancies, the different types of folate isoforms that are available and the potential consequences of over-supplementation.

## 2. Folate: Definition, Uptake and Function

Folate is a term for a family of essential, water-soluble micronutrients that, once converted to tetrahydrofolate (THF), play a pivotal role in the one-carbon-metabolism. As all animals are unable to synthesize folate, dietary intake is necessary [12]. Natural folates can be found in the polyglutamate form in leafy green vegetables, lentils, beans and citrus fruits. These folates need to be hydrolyzed to the mono-glutamate form by folate conjugase in the intestinal mucosa in order to be transported through the intestinal wall. This conversion is a pH-sensitive process with an optimum between 5.5 and 6.0, which indicates that factors which alter the intestinal pH can influence conversion and absorption rates of folate [12,13,14]. Absorption occurs mainly through active transport by a proton-coupled transporter protein that is expressed mainly in the brush border cells of the duodenum and proximal jejunum, with passive transport becoming more important at higher doses of folate [15].

**Figure 1:**
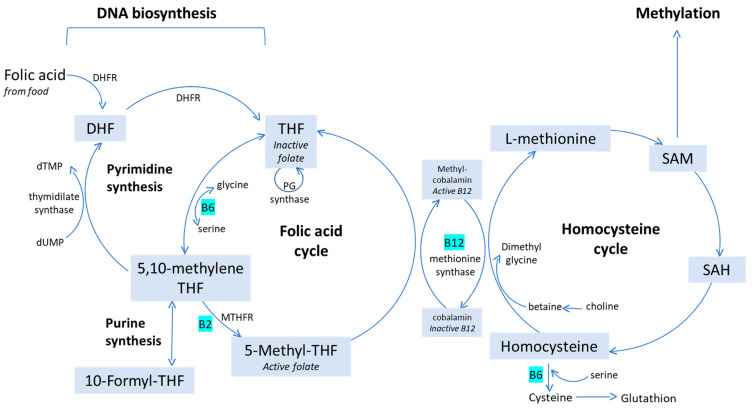
Folate-mediated metabolism and connection with homocysteine cycle.

Folic acid (FA), a synthetic precursor compound that does not exist in nature, has the advantage of being more stable than reduced folates. However, FA itself is not an active coenzyme, meaning that it has no biological function prior to being reduced by dihydrofolate reductase (DHFR) to dihydrofolate (DHF) and ultimately THF (Figure 1). THF can either be converted into 10-formyl-THF by methyl-THF dehydrogenase (MTHFD), after which it will act as a carbon-group donor in the synthesis of purine, or it can be transformed into 5,10-methylene-THF by serine hydroxy-methyltransferase (SHMT). This molecule can be used in two ways: (1) as a co-factor in the conversion of dUMP into dTMP by thymidylate synthase, and will as such play a role in the synthesis of pyrimidine—in this reaction, 5,10-methylene-THF is transformed into DHF which in turn is transformed back in THF by DHFR; (2) as 5-methyl tetrahydrofolate (5-MTHF) after conversion by the enzyme methylene tetrahydrofolate reductase (MTHFR). Once available, 5-MTHF donates a methyl group to homocysteine, forming methionine under the influence of methionine synthase, with vitamin B12 as a co-factor. Methionine can be trans-methylated into S-adenosylmethionine (SAM) and will then act as the most important methyl-group donor in the cell, playing a role in the methylation of DNA, histones and other proteins [12]. Once 5-MTHF has donated a methyl-group to homocysteine, it is converted back to THF [12,14].

As folate plays a pivotal role in both methylation, purine synthesis and pyrimidine synthesis, one can understand that deficiencies or alterations in the folate metabolism can affect development, gene expression and genomic stability, even more so when cell division is occurring rapidly (e.g., hematopoiesis, fetal development).

## 3. Folate-Status in Non-Bariatric Pregnancies

For women without a personal history of pregnancy affected by neural tube defects, several national guidelines, e.g., NHS in the UK and the US Preventive Services Task Force, recommend using a supplement containing 400 µg folate per day periconceptional in order to reduce the risk for neural tube defects [16]. Based on a systematic review by Blumfield et al., compromised of 62 studies in pregnant women from developed countries, folate intake during pregnancy varied between 13% and 63% below recommendations in all regions [17]. In the GLIMP2 Study it was shown that, even in a highly educated population of Dutch women who wished to become pregnant, 50% of the women did not meet the recommended intake of folate and 14% of the women had an inadequate folate status [18]. A study of healthy ethnic Danish pregnant women suggests that the increased requirements of folate during pregnancy may increase the risk of a low folate status in late pregnancy and postpartum, despite finding an adequate dietary folate intake in non-pregnant women of reproductive age [19]. Additionally, in the Boston Birth Cohort involving 7612 mothers from an urban low-income population, the proportion of mothers taking daily folic acid supplementation during preconception was only 4.3%, which rose to 55.9%, 59.4% and 58% during first, second and third trimester. Moreover, they found plasma levels of folate to be either too low or too high in approximately one third of mothers [16]. Similarly, in a cross-sectional analysis of 1003 pregnant US women, predominantly non-Hispanic white and at above 185% of the income-to-poverty ratio, a significant number were not meeting recommendations, while many were at risk of excessive consumption of folic acid [20]. As such, folate supplementation varies wildly in the population of women of reproductive age.

## 4. Folate Malabsorption after Bariatric Surgery: Pathogenesis and Prevalence

The absorption of folate is a delicate process, which is easily influenced by various factors. After bariatric surgery, two main aspects can be identified that can cause malabsorption: alterations in the intestinal pH, and decreased uptake capacity due to bypassing of the main absorption site.

As we have discussed earlier, the hydrolyzation of folate in the polyglutamate form to the mono-glutamate form is a pH-sensitive process with an optimum between 5.5 and 6.0. In a non-surgical patient, the intestinal pH is influenced by the gastric pH, which ranges from 1 to 3 due to gastric hydrochloric acid pumps. After various types of bariatric surgery, the stomach is reduced to a small pouch, limiting the number of pumps and increasing gastric pH, which in turn can increase intestinal pH to beyond the optimal range of hydrolyzation.

Secondly, the main mechanism for the uptake of folate is an active transport via a proton-coupled folate transporter protein. This protein is expressed in the brush border membrane of the duodenum and proximal jejunum [15], and as such is bypassed in RYGB or BPD. However, there are adaptive mechanisms which make it possible to absorb folate along the entire small intestine [21]. As the rate of absorption is unknown, screening for folate deficiencies remains important in patients after bariatric surgery. This is further supported by a statement of Via et al. [22], who demonstrated that 10–20% of bariatric patients experienced folate deficiencies following surgery. In contrast to this, a meta-analysis by Weng et al. [23] showed no increase in folate deficiencies 12 months after RYGB-surgery. The authors did state that deficiencies are correctable by regular supplementation. Another review by Lewis et al. [24] found reports on folate deficiency after SG, with eight studies reporting no significant change in prevalence at 12 months compared to baseline and one study reporting a decrease in deficiencies. The same review reported on five studies comparing folate deficiencies over 12 months after RYGB: four of these found no significant difference compared to baseline and one study reported a decrease in deficiency prevalence. The authors did note that the quality and limitations of the available research provided insufficient evidence to inform about the true prevalence of these deficiencies, as there was a large variation in reported reference ranges and a wide range of possible confounders influencing these results.

Folate status during pregnancies after bariatric surgery remains a highly relevant and underexplored issue. In a study that assessed birth outcomes after bariatric surgery, there were indications of nutritional deficiency in iron, vitamin B12 and folate [25]. Devlieger et al. also concluded in a multicenter prospective cohort study that women who underwent bariatric surgery showed frequent low micronutrient levels that could be potentially harmful for the women themselves and their offspring [26].

## 5. Types of Folate Supplementation: Advantages and Disadvantages of the Different Forms

As stated, folic acid is a precursor compound that has no biological function until it is reduced to dihydrofolate and THF. On the contrary, 5-MTHF is the biologically active form of folate and is the form present in circulation. In addition, it is the only folate able to cross the blood–brain barrier and plays a role as methyl group donor in the one-carbon metabolism, being involved in important cell functions such as DNA synthesis and methylation of homocysteine to methionine.

Supplemental 5-MTHF supports plasma folate more actively than FA in the general population and in MTHFR single nucleotide polymorphism (SNP) carriers [27]. In this mutant group, a DNA sequence variation in the MTHFR gene leads to the replacement of the single cytosine with thymine at nucleotide 677 (C677T polymorphism). In Europe, 12% of the population is homozygous (TT), 43% heterozygous (CT) and 45% wild-typed (CC) for this polymorphism [28]. The capacity to generate 5-MTHF can decrease by 17–75% in the C677T mutation (MTHFR block). The homozygous TT MTHFR isoform is detrimental to fertility and alters pre-implantation development, possibly also inducing chromosomal abnormalities [27]. For couples with fertility problems a physiological dose of 800 µg 5-MTHF bypasses the MTHFR block caused by the C677T mutation and is an effective treatment. A dose of 5-MTHF not exceeding 1 mg appears to be the best possible supplement composition in this respect for several reasons.

Firstly, the use of 5-MTHF instead of folic acid can eliminate the influence of the extremely slow and variable activity of DHFR and the effects of the MTHFR polymorphism. Bailey et al. concluded that the activity of DHFR becomes limiting when FA is consumed at levels higher than the tolerable upper intake level (1 mg/day for adults) [29]. It was shown that the low activity of DHFR in human liver is the main cause of temporary, high levels of unmetabolized folic acid (UMFA) in plasma. Elevated UMFA (UMFA syndrome) is suspected of causing immune dysfunction and other adverse pathological effects such as the progression of precancerous lesions in the colon or prostate [27]. MTHFR is a rate-limiting enzyme in the endogenous synthesis of 5-MTHF from folic acid. As explained earlier, the genetic variants of MTHFR are known to further limit the formation of 5-MTHF, impacting the methylation processes. Therefore, SNP carriers of the homozygous (TT) and heterozygous (CT) type are likely to benefit from supplemental 5-MTHF versus folic acid, since 5-MTHF is the predominant natural form that is readily available for transport and metabolism.

Secondly, 5-MTHF supplementation at least equals the effect of folic acid supplementation on serum and red blood cell (RBC) folate levels. In a randomized, placebo-controlled, double-blind trial, Venn et al. compared 5-MTHF (113 µg/d) and folic acid (100 µg/d) supplementation for 24 weeks in women of childbearing age (18–49 y). The results suggested that 5-MTHF and folic acid supplementation increased plasma folate and RBC folate levels to a similar extent [30]. In another double-blind clinical trial comparing the effects of folic acid (1 mg/day) vs. 5-MTHF (1 mg/day) in the management of idiopathic recurrent miscarriage, 5-MTHF increased serum folate concentration significantly more than the same amount of folic acid. However, there was no significant difference in serum homocysteine concentration and early spontaneous abortion rate between the two groups [31]. Lamers et al. showed that administration of 5-MTHF (416 µg/day) for 24 weeks improved RBC folate concentration more efficiently compared to the equimolar amount of folic acid (400 µg/day), indicating that 5-MTHF might be an efficient and safe alternative to folic acid [32]. In a meta-analysis comparing the strength of associations between total folate intake and folate status, Berti et al. observed that the relationship with folate status tended to be stronger for 5-MTHF than for folic acid. It was noted that 5-MTHF was equal to or more effective than folic acid in preserving folate status given that 5-MTHF is the predominant folate transport and storage form within the body [33]. Additionally, Prinz-Langenohl et al. evaluated the effect of a single oral dose folic acid (400 µg/day) vs. equimolar amount of 5-MTHF in healthy women stratified by the genotype of the C677T MTHFR mutation in a randomized double-blind crossover trial. Based on pharmacokinetic variables, 5-MTHF increased plasma folate more efficiently than folic acid in both genotypes. In this study UMFA in plasma occurred regularly after supplementation with folic acid, but only occasionally with 5-MTHF [28]. Finally, a 16-week randomized controlled trial by Houghton et al. compared the effect of 5-MTHF (416 µg/day), folic acid (400 μg/day) or placebo on blood folate indices in healthy lactating women. They found that the mean RBC folate concentration after supplementation with 5-MTHF was significantly higher than with folic acid or placebo, indicating that 5-MTHF was as effective as and even slightly better than folic acid in preserving RBC folate concentrations during lactation [34].

Thirdly, 5-MTHF is the main folate form in umbilical cord blood, and the concentration of 5-MTHF in cord serum has been shown to be roughly twice as high as in maternal serum, suggesting that supplementation with 5-MTHF during pregnancy can provide an immediate source of folate to be transported to the fetus [35]. In short, these studies demonstrate that 5-MTHF can effectively increase serum or blood folate markers. Therefore, supplementing with 5-MTHF for neural tube defect prevention seems to be a pragmatic approach to ensure maternal folate adequacy regardless of the potential presence of polymorphisms, and to reduce the risk of UMFA syndrome from folic acid supplementation.

Finally, a major advantage of 5-MTHF over folic acid is the reduced risk of concealing hematological symptoms of vitamin B12 (cobalamin) deficiency [12]. Both folate and vitamin B12 deficiencies are characterized by megaloblastic anemia, and both deficiencies lead to elevated blood homocysteine. In severe vitamin B12 deficiency, the B12-dependent methionine synthase enzyme is inactivated. As a result, cytosolic folate is trapped as 5-MTHF at the expense of other folate coenzyme forms required for one-carbon metabolism, such as thymidylate synthesis, which leads to a functional folate deficiency in the cell. The defective DNA synthesis seen in megaloblastic anemia is caused by an induced secondary folate deficiency (the ‘methyl trap’ hypothesis). Unlike 5-MTHF, folic acid is reduced directly to THF and ‘escapes’ the metabolic block caused by insufficient cobalamin (see Figure 1). Thus, folic acid supplementation potentially corrects the megaloblastic anemia caused by cobalamin deficiency by restarting DNA synthesis for RBC production, but it does not correct the underlying cobalamin deficiency. As a result, the hematological marker of the deficiency (the anemia) is corrected and the clinical sign of the deficiency is masked by the folic acid treatment. The resulting delay in diagnosis of the ongoing cobalamin deficiency can lead to irreversible neurological damage [36,37].

## 6. Folic Acid Over-Supplementation: Potential Consequences

Potential negative effects of high doses of folic acid have been reported. For example, Valera-Gran et al. observed that children whose mothers used folic acid supplements in doses higher than 5000 µg/d during pregnancy had a statistically significant lower mean psychomotor scale score compared to children whose mothers used a more general recommended dose of folic acid supplements (400–1000 µg/day) [36]. In a prospective study evaluating the association between maternal multivitamin supplementation, maternal plasma folate and vitamin B12 levels at birth and offspring Autism Spectrum Disorder (ASD) risk in 1257 mother-child pairs, Raghavan et al. reported that low (≤2 times/week) and high (>5 times/week) supplementation was associated with increased risk of ASD. In mothers with very high levels of plasma folate (≥60.3 nmol/L) due to high folic acid intake, resulting in an accumulation of folic acid, as well as very high levels of plasma B12 (≥536.8 pmol/L) at birth, a 2.5 times increased risk of ASD in offspring was observed. This study thus illustrated the concern about extremely high levels of plasma folate and B12 exposure in-utero on early brain development [38]. Wiens and DeSoto advised similar caution regarding the fact that unexpectedly high levels of FA may have implications for proper methylation of DNA during times of rapid cell division, for example, in prenatal development. Whilst the prevalence of neural tube defects has diminished significantly since the start of folic acid fortification in North America in 1998, continued high levels of FA supplementation throughout a pregnancy may not be needed, and are not without risk [39]. In this respect, national folic acid food fortification programs were also challenged by Murphy and Westmark [40].

It has been suggested that a combination of a diet rich in folate and supplementation with folic acid during pregnancy may also have potential adverse outcomes. As indicated in the Norwegian Mother and Child Cohort, risk of asthma in children was increased in the highest versus lowest quintile of total folate intake in mothers during pregnancy [41].

Lastly, periconceptional maternal intake of methyl-group donors such as folic acid and folate can provoke epigenetic changes in genes related to metabolism, growth and appetite control in the offspring. For instance, Pauwels et al. observed changes in methylation in children exposed to a higher maternal intake of methyl-group donors [42].

## 7. Folate and Fetal Growth

Intra-uterine growth restriction (IUGR) is a common complication in pregnancies after bariatric surgery, with the odds ratio being doubled when compared to controls with similar pre-surgery or pre-pregnancy BMI [9,43]. The exact pathophysiological mechanism of this has not yet been fully revealed, but micronutrient deficiencies have been noted as a possible contributing factor. In the case of folate, past research has demonstrated a correlation between decreased maternal plasma levels and the risk for fetal growth restriction. An Australian prospective observational cohort including 137 subjects found that RBC folate levels at mid pregnancy were a strong predictor of IUGR later [44]. Similarly, Lindblad et al. prospectively followed 128 Pakistani women and found an increased occurrence of IUGR when maternal and cord concentrations of folate were low [45]. In contrast to these findings, it is unclear whether folate supplementation improves these outcomes. Data from the Screening for Pregnancy Endpoints (SCOPE) cohort, compromised of 3196 nulliparous women, showed that 11.3% of women who did not take a folic acid supplement had an SGA-infant, whilst this condition was found in 9.3% and 8.3% of women taking <800 µg and ≥800 µg of folic acid, respectively. Although a tendency towards improvement of this outcome can be noticed, these findings failed to reach significance [46]. In a secondary analysis of this cohort, Bulloch et al. examined maternal single nucleotide polymorphisms related to folate metabolism, folic acid supplement use and the association between these and SGA [47]. They found a significant interaction between three polymorphisms (THFR 677, MTHFR 1298, and TCN2 776) and folic acid supplementation, in which supplementation attenuated the risk of developing SGA versus non-supplemented controls. This does stress the importance of providing adequate folate in these populations. Lastly, there is some evidence regarding an altered placental folate transport in pregnancies affected by IUGR. Chen et al. investigated the placentas of women delivering an IUGR-infant and placentas of those delivering an normal weight baby [48]. Folate transporter expression was measured and the Reduced Folate Carrier (RFC) was found to be significantly less prevalent in the IUGR group (the Folate Receptor-α was not affected). As such, folate uptake was found to be significantly lower in the placental microvillus plasma membrane of patients affected by IUGR (−38%, *p* < 0.05). The authors propose that continued supplementation of folate could be beneficial in pregnancies affected by IUGR, as this can aid in improving fetal folate availability despite the placental transport defect.

Based on the discussed findings, it seems reasonable to continue folate supplementation beyond the first trimester in pregnancies after bariatric surgery, as the risk for IUGR is increased and might be in part attenuated by increasing the bioavailability of folate.

## 8. Conclusions and Recommendations

The folate status of pregnant mothers may have relevant health consequences for the mother herself and the developing child. In addition to the well-documented association between folate supplementation and prevention of neural tube defects, supplemental folate was also associated with higher live birth rates after assisted reproductive technology treatment and reduced risks of spontaneous abortion and congenital heart defects [49,50,51]. Conversely, detrimental effects of high dosages of folic acid supplements on child psychomotor development, offspring ASD and childhood asthma have been documented [38,40,41,52].

In a post-bariatric surgery setting, general guidelines for this high-risk pregnancy group have not been clearly substantiated, although many clinics have put forward recommendations. The most important is the suggestion to conceive after losing maximum weight, resulting in the recommendation to wait 12–24 months post-surgery. Unfortunately, the recommended intake for folate for bariatric patients planning a pregnancy is not consistent. Several national guidelines, such as those issued by the British Obesity & Metabolic Surgery Society (BOMMS), advise a supplement containing 4 to 5 mg of folate per day for patients with obesity in the periconceptional period. In practice, these national guidelines are often being applied to bariatric patients even though these women have a statistically significantly lower pre-pregnancy BMI compared to the obese pregnant controls (28.1 ± 5.1 versus 34.3 ± 3.7 kg/m^2^, *p* < 0.001) [53,54]. Xanthakos et al. also observed no significant changes in serum levels of folate between baseline and 5 years after laparoscopic bariatric procedure in the 242 adolescents (13–19 years old) enrolled in the Teen-LABS cohort study [55]. Therefore, a critical review of folate requirement in pregnancy post-bariatric surgery is needed.

The use of high dose folic acid supplements should be discouraged in order to avoid UMFA in the circulation and its potential negative effects. Our recommendation is in line with the recent clinical practice guidelines for perioperative nutrition, metabolic and nonsurgical support of patients undergoing bariatric procedures (cosponsored by the American Association of Clinical Endocrinologists/American College of Endocrinology, the Obesity Society, American Society for Metabolic & Bariatric Surgery, the Obesity Medicine Association and American Society of Anesthesiologists), which states that supplementation with folate in a dose of more than 1 mg per day is not recommended because of the potential masking of vitamin B12 deficiency [56]. Timing, on the other hand, is of the utmost importance, since Crider et al. demonstrated that stable levels of folate RBC are achieved after 9 months for those consuming a 375–570 µg/day dose. Moreover, the lowest risk of NTDs is seen in patients with a prolonged intake [57].

In conclusion, the use of a supplement containing 400 µg 5-MTHF in the periconceptional period and during early pregnancy is supported by the existing literature for bariatric patients without any precedent of neural tube defects. Most importantly, when there are no specific medical needs for a high dose of folate, a supplement should not exceed a daily dose of 1mg folate during pregnancy and in a post-bariatric setting.

## Data Availability

The study did not report original data.
